# Distributed Query Plan Generation Using Multiobjective Genetic Algorithm

**DOI:** 10.1155/2014/628471

**Published:** 2014-05-14

**Authors:** Shina Panicker, T. V. Vijay Kumar

**Affiliations:** ^1^SFIO-NIC Division, National Informatics Center, Ministry of Information Technology, New Delhi 110003, India; ^2^School of Computer and Systems Sciences, Jawaharlal Nehru University, New Delhi 110067, India

## Abstract

A distributed query processing strategy, which is a key performance determinant in accessing distributed databases, aims to minimize the total query processing cost. One way to achieve this is by generating efficient distributed query plans that involve fewer sites for processing a query. In the case of distributed relational databases, the number of possible query plans increases exponentially with respect to the number of relations accessed by the query and the number of sites where these relations reside. Consequently, computing optimal distributed query plans becomes a complex problem. This distributed query plan generation (DQPG) problem has already been addressed using single objective genetic algorithm, where the objective is to minimize the total query processing cost comprising the local processing cost (LPC) and the site-to-site communication cost (CC). In this paper, this DQPG problem is formulated and solved as a biobjective optimization problem with the two objectives being minimize total LPC and minimize total CC. These objectives are simultaneously optimized using a multiobjective genetic algorithm NSGA-II. Experimental comparison of the proposed NSGA-II based DQPG algorithm with the single objective genetic algorithm shows that the former performs comparatively better and converges quickly towards optimal solutions for an observed crossover and mutation probability.

## 1. Introduction

Advancement in technology has made it possible today to gather timely and effective information from vast sources of data (sites) distributed geographically across a network. The users at local sites can work independently as well as communicate with other sites to retrieve data for answering global queries. Such a setup is referred to as a Distributed Database System (DDS) [1, 2]. Query posed on a DDS is generally decomposed into subqueries, which are processed at the respective local sites where the data resides, before being transmitted to another site for cumulative processing of distributed data fragments. At the user end, an integrated result is displayed. A distributed query processing strategy aims to minimize the overall cost of query processing in such systems [3]. The cost of query processing in a DDS comprises of two costs: the local processing cost and the site-to-site communication or the transmission cost of relation fragments. The total cost incurred in processing a distributed query can thus be taken as the sum of the local processing cost at the individual participating sites and the cost of data communication among these sites. Local processing cost comprises of the cost of join operation on relations accessed by the user query and the communication cost is proportional to the size of relation fragments being transmitted among sites and its cost of transmission. These costs need to be minimized in order to minimize the total query processing cost.

In today's scenario, with a multifold increase in the size of DDS, the communication cost asserts a major impact on the overall cost of query processing. The cost incurred in communicating data through a congested network path or the communication of large data units between sites with higher communication costs can highly influence the cost of query processing and thus the sequence of sites through which the data fragments get processed has a significant impact on the overall query processing cost. It thus also plays a key role in determining the overall performance of a DDS. There can be a number of possible ways to process and communicate relation fragments involved in the query. A distributed query processing strategy evaluates all possible sequence of sites corresponding to relations accessed in the query, referred to as a query plan, and determines the most optimal query plan that minimizes the total cost that is local processing (CPU, I/O) cost and communication cost [5–11]. The number of possible query plans grows at least exponentially with the increase in number of relations accessed by the query [12, 13]. This number increases further if relations accessed by the query are replicated across multiple sites. Performing an exhaustive search on all possible combinations of query plans is not feasible due to a vast search space. Therefore, in large DDS, devising a query processing strategy that optimizes the total query processing cost is shown to be a combinatorial optimization problem [10].

Over the last three decades, many algorithms and techniques have been devised to solve the class of combinatorial optimization problems. Initially, the rigorous mathematical and search based techniques like simulated annealing, random search algorithms, dynamic programming, and so forth were used to solve such problems, which though worked well with moderate sized problems on cost heuristic could not succeed with complex multiobjective problems. These mechanisms suffered from a drawback at certain instances, where they converged to local optima without exploring the entire search space [8, 13, 14]. However, in the last two decades, evolutionary techniques have gained immense popularity due to their applicability in solving these complex scientific and engineering optimization problems. These algorithms are inspired by the Darwinian evolution that accentuates the concept of  “Survival of the Fittest” [15]. It is, thus, metaphorical to the natural social behavior and biological evolution of species. The evolutionary techniques are now proved to be the most proficient method of choice for solving such problems. Genetic algorithm based techniques which belong to the class of evolutionary algorithms have also been widely used in solving complex real life science and engineering problems. The strength of GA as a metaheuristic comes from its ability to combine the good features from several solutions to create new and better solutions [16, 17] over generations.

Most real world scientific and engineering problems have often conflicting and competing objectives that need to be optimized. The evolutionary strategies are proved to be best suited for this class of problems as they can simultaneously optimize the different objectives and find efficient tradeoffs unlike the classic techniques, where the objectives were separately optimized and weighed based on the prior knowledge about the problem in hand. The first pioneering study on multiobjective evolutionary optimization came out in mideighties [18]. In subsequent years, several different evolutionary algorithms (VEGA [19], MOGA [20], NPGA [21], NSGA [22], NSGA-II [4], SPEA [23], SPEA-II [19], PAES [24], PESA [25]) have successfully been implemented to solve the classic optimization problems, for example, the single source shortest path problem [26], the all-pairs shortest path problem [27], the multiobjective shortest path problem [28], the travelling salesman problem [29], the knapsack problem [30], and so forth. Recently, new evolutionary techniques, for example, particle swarm optimization [31], artificial immune systems [32], frog leaping algorithm [33], ant colony optimization [34], and so forth, have been successfully applied to the multiobjective optimization paradigm.

This paper addresses the distributed query plan generation (DQPG) problem given in [3]. This problem is based on a heuristic that favors query plans involving less number of sites participating to retrieve the results. Further, query plans involving smaller relations transmitted over less costly communication channels would incur less communication costs and are thus favored over others. Query plans generated based on this heuristic would result in efficient query processing. This DQPG problem was formulated and solved as a single objective optimization problem in [3]. Since this DQPG heuristic comprises minimization of both the local processing cost and the communication cost, an attempt has been made in this paper to minimize these costs simultaneously. That is, the DQPG problem is formulated as a biobjective optimization problem comprising two objectives, namely, minimization of the total local processing cost and minimization of the total communication cost. In this paper, this problem has been solved using the multiobjective genetic algorithm NSGA-II (nondominated sorting genetic algorithm) [4]. The proposed NSGA-II based DQPG algorithm attempts to simultaneously minimize the two objectives with the aim of achieving an acceptable tradeoff amongst them. It is shown that the optimization of total query processing cost using the proposed algorithm gives considerable improvement with respect to the time taken to converge and the quality of solutions, with respect to total query processing cost, when compared to the single objective GA based DQPG algorithm given in [3].

This paper is organized as follows.  Section 2 discusses the DQPG problem and its solution using the simple genetic algorithm (SGA) given in [3].  Section 3 discusses DQPG using the multiobjective genetic algorithm. An example illustrating the use of the proposed NSGA-II based DQPG algorithm for generating optimal query plans for a distributed query is given in Section 4. The experimental results are given in Section 5.  Section 6 is the conclusion.

## 2. DQPG Using SGA

This paper addresses the DQPG problem given in [3], solved using SGA. The DQPG problem is discussed next followed by a brief example describing the underlying methodology.

### 2.1. The DQPG Problem

Query plan generation is a key determinant for the efficient processing of a distributed query. This necessitates devising a query plan generation strategy that would result in efficient query processing. This strategy would require minimizing the total cost of query processing. The total cost incurred comprises the joint cost that is the cost incurred in processing the query locally at the individual sites and the cost of communicating the relation fragments among the sites. A distributed query processing strategy is given in [3], which aims to minimize the total query processing cost (TC) given below [3]:

(1) $$\begin{array}{c} \text{TC}=\overset{m}{\underset{i=1}{\sum }}\text{LP}{\text{C}}_{i}\times a_{i}+\overset{i=m-1,\;j=m}{\underset{i=1,\;j=i+1}{\sum }}\text{C}{\text{C}}_{ij}\times b_{i}, \end{array}$$

where LPC_*i*_ is the local processing cost per byte at site *i*, CC_*ij*_ is the communication cost per byte between sites *i* and *j*, *a*_*i*_ is the bytes to be processed at site *i*, *b*_*i*_ is the bytes to be communicated from site *i*, and *m* is the total number of sites. For each relation *R*_*t*_, Card(*R*_*t*_) represents its cardinality and Size(*R*_*t*_) represents the size of a single tuple in bytes. At each site, the relations are integrated on common attributes using the equijoin operator to arrive at a single relation [3].

For relations *R*_*t*_, with cardinality Card(*R*_*t*_) and *R*_*s*_ with cardinality Card(*R*_*s*_) at site *i*, the cardinality Card_*i*_ of the resultant relation *R*_*i*_ is given as [3]

(2) $$\begin{array}{c} \text{Car}{\text{d}}_{i}=\frac{\text{Card}\left(R_{t}\right)\times \text{Card}\left(R_{s}\right)}{\text{Dis}{\text{t}}_{ts}\times \min\left(\text{Card}\left(R_{t}\right),\text{Card}\left(R_{s}\right)\right)}, \end{array}$$

where Dist_*ts*_ is the number of distinct tuples in the smaller relation among *R*_*t*_ and *R*_*s*_.

The size of the resultant relation *R*_*i*_ at site *i* is given as [1, 3]

(3) $$\begin{array}{c} \text{Siz}{\text{e}}_{i}=\text{Size}\left(R_{t}\right)+\text{Size}\left(R_{s}\right). \end{array}$$

For a given query plan, the communication between sites occurs in the order starting from the site having a relation with lower cardinality to the site having a relation with higher cardinality [3]. The communication cost CC_*ij*_ and local processing cost LPC_*i*_ are known a priori.

The number of bytes to be processed locally at site *k* is given by *a*_*k*_ [3]:

(4) $$\begin{array}{c} a_{k}={\text{Card}}_{i}\times {\text{Size}}_{i}. \end{array}$$

The number of bytes to be communicated from site *j* to site *k* is given by *b*_*j*_ [3]:

(5) $$\begin{array}{c} b_{j}=\text{Car}{\text{d}}_{j}\times \text{Siz}{\text{e}}_{j}. \end{array}$$

Distributed query plans based on the above heuristic is generated using simple GA (SGA) in [3]. This SGA based DQPG, as given in [3], is discussed next.

### 2.2. SGA Based DQPG

As discussed above, it is a very complex task to generate efficient query plans from among a large set of possible query plans. An SGA based DQPG strategy, based on the heuristic defined above, is given in [3], which aims to minimize the total cost of query processing (TC) indicating the fitness of a particular solution as compared to others in the population. The algorithm considers relations accessed by the query, crossover and mutation probability, and the prespecified number of generations (*G*), as input, and produces the Top-*K* query plans as output. First, the algorithm randomly generates an initial population of valid query plans (chromosomes), where the size of a query plan is equal to the number of relations accessed by the query. Each gene in a chromosome represents a relation and the ordering of relations in a chromosome is in increasing order of their cardinality. The value of a gene is the site where the corresponding relation resides. As an example, for a query accessing four relations (*R*1, *R*2, *R*3, and *R*4) arranged in the increasing order of cardinalities, one of the encoding schemes for the chromosome representation can be (1, 1, 4, 3) implying that *R*1 and *R*2 are in site 1, *R*3 is in site 4, and *R*4 is in site 3. The fitness (TC) value is computed for each of the query plans and thereafter the query plans are selected for crossover using the binary tournament selection technique [35]. These selected query plans undergo random single-point crossover [15, 36], with probability *P*_*c*_, and mutation [15, 36], with probability *P*_*m*_. The resultant new population replaces the old population and the above process is repeated for the prespecified number of generations *G*. Thereafter, the Top-*K* query plans are produced as output. In this paper, the above single objective DQPG problem is formulated and solved as a multiobjective DQPG problem as will be discussed next.

## 3. DQPG Using Multiobjective Genetic Algorithm

In this paper, the single objective DQPG problem discussed above is formulated as a biobjective DQPG problem. This formulation is given next.

### 3.1. Multiobjective DQPG Problem Formulation

In the GA based DQPG algorithm given in [3], there is a single objective, that is, Minimize TC. It can be observed that TC comprises two costs, namely the local processing cost incurred at participating sites, that is, total processing cost (TPC), and communication cost between the participating sites, that is, total communication cost (TCC). Since minimizing TC would require minimizing TPC and minimizing TCC, this single objective (Minimize TC) DQPG problem is formulated as a biobjective DQPG problem comprising two objectives as* Minimize *TPC and* Minimize *TCC. Consider

(6) $$\begin{array}{c} \text{TCC}=\overset{i=u-1,\;j=u}{\underset{i=1,\;j=i+1}{\sum }}{\text{CC}}_{ij}\times b_{i},\\ \text{TPC}=\overset{u}{\underset{i=1}{\sum }}{\text{LPC}}_{i}\times a_{i}, \end{array}$$

where *u* is the number of sites accessed by the query plan in ascending order of cardinality per site, CC_*ij*_ is the communication cost per byte between sites *i* and *j*, LPC_*i*_ is the local processing cost per byte at site *i*, *b*_*i*_ is the bytes to be communicated from site *i*, and *a*_*i*_ is the bytes to be processed at site *i*. CC_*ij*_, LPC_*i*_, *b*_*i*_, and *a*_*i*_ are as discussed in Section 2.1. If a site contains a single relation, its LPC is considered zero. TCC and TPC need to be minimized simultaneously to achieve an acceptable tradeoff.

The above multiobjective DQPG problem has been solved using the multiobjective genetic algorithm, which is discussed next.

### 3.2. Multiobjective Genetic Algorithms

Conceptualization of multiobjective problems using veridical models has a great resemblance to many real world engineering and design problems that involve more than one coextensive and often competing objectives, that is, maximize profit, maximize throughput, minimize cost, minimize response time, and so forth. In such a scenario, no single solution can be termed as optimal, as in the case of single objective optimization problems, but rather a set of alternative solutions can be visualized as a tradeoff between the different objectives under consideration. This set of solutions is regarded superior to others in the search space, as no other recorded/available solution can better optimize all the objectives considered together [37–39].

Multiobjective optimization approaches can be broadly classified into three categories [37]. The approaches in the first two categories can be termed as the classical optimization approaches, which combine all objectives into a single composite function using some combination of arithmetic operators or move all but one objective into the constraint set. The approaches in the first category have limitations in regard to appropriate selection of weights and designing functions in accordance to the problem. It would mandate the user to have a priori knowledge of the behavior of each objective function to some extent for providing the range of values to objectives so that none of them dominate the others, which is not always possible [17]. This approach is generally denominated as aggregating functions and it has been implemented at several occasions with relative success in situations where behavior of the objective function is more or less well-known. Some of the aggregating functions include the weighted sum approach, goal programming, *ε*-constraint method, and so forth [40]. In the second approach, moving the objectives into a constraint set requires that the boundary values for each of the objectives be known a priori, which is almost impossible. In either of the two cases, the optimization method returns a single solution rather than a set of solutions, giving possible tradeoffs; and therefore the quality of solution in these approaches greatly depends upon the correct problem formulation. If feasible, these would be the most efficient and simplest approaches, which would give, atleast, sub optimal results in most cases.

The third approach overcomes the problems faced in the classical optimization approaches and emphasizes the development of alternative techniques based on exploring the complete set of nondominated solutions and thereby enabling the decision maker to choose among the different alternatives. This set of solutions is referred to as the Pareto optimal set [13]. A Pareto optimal set can be formally defined as a set of solutions that are nondominated with respect to each other, that is, replacing one solution with another, within the Pareto optimal set, will invariably lead to a loss to one objective against a gain obtained in another objective [41]. Pareto optimal sets can have varied sizes but usually the size increases with increase in the number of objectives [37, 40]. They are more preferred over single solutions as they closely resemble real world problems, where the decision maker makes a decision based on tradeoffs between multiple objectives. A number of techniques were formulated to generate the Pareto optimal set, for example, simulated annealing [14], Tabu search [42], ant colony optimization [34], and so forth. The problem with these algorithms was that most often they get struck at local optima and thus render it infeasible to venture out for identifying new tradeoffs. Evolutionary algorithms such as GA, on the other hand, seem to be especially suited for this task as they enable parallel exploration of different areas in the search space, eventually exploiting the solutions attained using operators such as crossover and mutation [13]. It would enable determining more members of a Pareto optimal set in a single run instead of a series of runs required in other blind search strategies. Also, the evolutionary algorithms require very little a priori knowledge of the problem at hand and therefore are less susceptible to the typical shape and continuity of the Pareto front. The Pareto front can be defined as the points that lie on the boundary of the Pareto optimal region. These algorithms thus avoid convergence to a suboptimal solution [43].

Mathematically, a multiobjective optimization problem with *m* decision variables and *n* objectives can be defined without any loss of generality as a maximization or minimization problem given by [13, 38]

(7) $$\begin{array}{c} \text{minimize}=f\left(x\right)=\left(f1\left(x\right),f2\left(x\right),\ldots ,fn\left(x\right)\right),\\ \text{where}\;x=\left(x1,x2,\ldots ,xm\right)\in X\\ y=\left(y1,y2,\ldots ,yn\right)\in Y. \end{array}$$

Here, *x* is the decision vector, *X* refers to the parameter space, *y* is the objective vector, and *Y* defines the objective space. These objectives may be conflicting in nature, that is, improvement in one may lead to deterioration in another. So, it may become impossible to optimize all objectives simultaneously in a single solution. Instead, the best tradeoff solution would be of interest to a decision maker. These solutions form a Pareto optimal set which was initially coined by Edgeworth and Pareto and is formally defined as [13, 38].

“A decision vector *a*, is said to be Pareto optimal if and only if *a*, is nondominated regarding *X*. A decision vector *a*, is said to be nondominated regarding a set *X*′⊆*X*, if and only if there is no vector in *X*′ which dominates *a*. Formally it can be defined as Ǝa′∈X′:a′≺a”.

Also, a decision vector *a* ∈ *X* is said to dominate a decision vector *b* ∈ *X* (also written as *a*≺*b*), if and only if

(8) $$\begin{array}{c} \forall i\in \left\{1,2,\ldots ,n\right\}:f_{i}\left(a\right)\leq f_{i}\left(b\right),\\ \forall j\in \left\{1,2,\ldots ,n\right\}:f_{j}\left(a\right)\leq f_{j}\left(b\right). \end{array}$$

Several multiobjective algorithms exist in the literature [4, 18–25, 37, 40, 41, 44, 45] of which GA based multiobjective optimization algorithms have been widely used for solving multiobjective optimization problems. In this paper NSGA-II has been used to solve the DQPG problem. NSGA-II will be discussed next.

#### 3.2.1. NSGA-II

**Figure 1:**
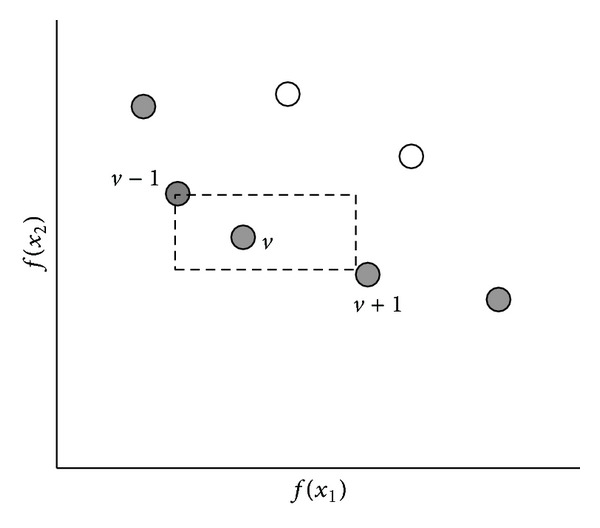
Crowding distance for solution “*v*” [4].

The basis of NSGA-II [4] lies in the nondominated sorting genetic algorithm (NSGA) introduced by Srinivas and Deb [22]. As the name suggests, NSGA uses nondominated ranking for each individual in the population and assigns them accordingly into nondominated fronts. The individuals in the first front or the nondominated individuals are then assigned large dummy fitness values. All individuals in the front shared this fitness value based on a sharing function. Next, the individuals in the second nondominated front are considered and similarly assigned a dummy fitness lower than the fitness assigned in the previous front. This process continues till the entire population is classified into fronts. Since the solutions in the first front have the maximum fitness value, their chances of selection increase and eventually more copies of such solutions get passed on to the next generation. However, NSGA suffered from some drawbacks such as high computational complexity *O*(*mN* ^3^), nonelitist approach, and the requirement of specifying a shared parameter [4]. These limitations were addressed in NSGA-II proposed by Deb et al. [4] as an improved version of NSGA [22]. It alleviates the drawbacks in NSGA by reducing the computational complexity to *O*(*mN* ^2^). Further, it uses a parameter-less sharing approach by using a crowding distance measure for selection. The crowding distance is an estimate of the density of solutions surrounding a particular solution in the objective space. In Figure 1, the crowding distance of solution represented as point *v* is computed as the average distance between the two closest solutions represented as points *v* − 1 and *v* + 1 on either side of the points *v* along each of the objectives *f*(*x*1) and *f*(*x*2).

NSGA-II uses a crowded-comparison operator for selection, which takes into account both the nondomination rank of a query plan in the population and its crowding distance. The nondominated solutions are preferred over dominated solutions and between two solutions having the same rank, a solution that resides in the less crowded region is preferred, that is, a solution for which the crowding distance is higher. The NSGA-II does not use any external memory but it ensures elitism by combining the best parents with the best offspring obtained [19]. In this paper an NSGA-II based multiobjective DQPG algorithm is used to compute optimal query plans for a given distributed query. This algorithm is discussed next.

### 3.3. NSGA-II Based DQPG Algorithm

The proposed NSGA-II based DQPG algorithm takes the relations given in the FROM clause of the distributed query as input. It arranges these relations in increasing order of their cardinalities. It then generates a fixed set of feasible query plans (chromosomes) based on the possible combinations of sites in which these relations are residing. Each gene in a chromosome represents a relation and is arranged in increasing order of the corresponding relation's cardinality. The value of a gene represents the site in which the corresponding relation resides. For example, suppose that a query posed by the user has 4 relations (*R*1, *R*2, *R*3, and *R*4) arranged in ascending order. The relation *R*1 is stored in sites *S*1 and *S*3, *R*2 is stored in *S*1, *R*3 is stored in *S*1 and *S*2, and *R*4 is stored in *S*1. Then the initial population of feasible query plans (chromosomes) can be (1, 1, 1, 1), (3, 1, 1, 1), (3, 1, 2, 1), and (1, 1, 2, 1). This defines the encoding scheme for the given problem. The proposed DQPG algorithm based on NSGA-II is given in Algorithm 1. The steps involved in this algorithm are discussed as follows.

**Step 1 (Initialize the Population [4, 46])**

A random population of query plans is generated as per the encoding scheme discussed above.

**Step 2 (Evaluate Query Plans on the Objective Functions)**

For each of the query plans in the population, the TCC and TPC values are computed as given below:

(9) TCC=∑i=1, j=i+1i=u−1, j=uCCij×bi,TPC=∑i=1uLPCi×ai,

where *u* is the number of sites accessed by the query plan in ascending order of cardinality per site, CC_*ij*_ is the communication cost per byte between sites *i* and *j*, LPC_*i*_ is the local processing cost per byte at site *i*, *b*_*i*_ is the bytes to be communicated from site *i*, and *a*_*i*_ is the bytes to be processed at site *i*. The procedure to compute CC_*ij*_, LPC_*i*_, *b*_*i*_, and *a*_*i*_ is given in Section 2.1. If a site contains a single relation, its LPC is considered zero. TCC and TPC need to be minimized simultaneously to achieve an acceptable tradeoff.

**Step 3 (Perform Nondominated Sort [4, 46])**

On the given population, a fast nondominated sorting is performed in the following manner.

Two objective functions are considered. The first objective is to minimize the total processing cost (TPC) and the second objective is to minimize the total communication cost (TCC). NSGA-II attempts to find a tradeoff between these two objectives that can result in minimum total query processing cost (TC).

In order to perform a nondominated sort, each query plan is compared with every other query plan in the population to find if it is dominated. For each query plan “*i*”, the following two entities are considered.

- *n*_*i*_: The number of query plans that dominate the query plan *i*.
- *S*_*i*_: The set of query plans that query plan *i* dominates.

All query plans that have *n*_*i*_ = 0 are added to the set Ŧ_1_. Set Ŧ = Ŧ_1_ where Ŧ is called the* current front*. For each element *x* in the current front, visit each member *j* in the set *S*_*x*_ and reduce the count of *n*_*j*_ by 1. Now if *n*_*j*_ gets reduced to zero for some *j*, add it to the set Ŧ_2_. After evaluating all the members of Ŧ in a similar manner, set Ŧ = Ŧ_2_. This process continues till all the query plans are assigned some front. The fast nondominated sorting procedure takes the current population as input and produces a list of nondominated fronts Ŧ_*i*_ as output.

**Step 4 (Density Estimation Using Crowding Distance [4, 46])**

After the nondominated sort, the crowding distance is computed for each query plan in Ŧ_*i*_. Crowding distance [4] is an estimate of the density of solutions surrounding a particular solution point in the population. It is defined as the average distance of the two closest points on either sides of the given point along each of the objectives. The crowding distance *I*(*distance*) is computed in the following manner [4, 46].

For each front Ŧ_*i*_, let *N* be the number of query plans in front Ŧ_*i*_. Initially the crowding distance for each query plan in the front Ŧ_*i*_ is zero. That is, *I*(*d*_*j*_) = 0; *j* = 1,…, *N*. Next, for each objective function, the query plans in the front Ŧ_*i*_ are sorted based on their value of TPC (i.e., the first objective function) and similarly also with respect to TCC (i.e., the second objective function) and placed in *I*(*distance*). The query plans having the smallest and the highest *I*(*distance*) values in both sets are assigned an infinite value for *I*(*distance*); that is, *I*(*d*_1_) = *∞* and *I*(*d*_*N*_) = *∞*. For remaining query plans, that is, *k* = 2,…, *N* − 1, *I*(*distance*) is computed as follows [4, 46]:

(10) $$\begin{array}{c} I\left(d_{k}\right)=I\left(d_{k}\right)+\frac{I{\left(k+1\right)}_{m}-I{\left(k-1\right)}_{m}}{f_{m}^{\max}-f_{m}^{\min}}, \end{array}$$

where *I*(*k*)_*m*_ is the value of *m*th objective function of *k*th query plan in front Ŧ_*i*_ and *f*_*m*_^max⁡^ and *f*_*m*_^min⁡^  are the maximum and minimum values obtained for the objective function *m*.

**Step 5 (Binary Tournament Selection)**

After assigning the crowding distance to the query plans in each front, a selection process is carried out. The selection scheme used is the binary tournament selection and it is carried out using the crowded comparison operator (≺_*n*_) [4, 46]. It uses two parameters as given below:

- **ρ**_rank⁡_ (nondomination rank). The query plans in front Ŧ_*i*_ will have **ρ**_rank⁡_ = *i*.
- *I*_*i*_(*d*_*j*_) (crowding distance in front *i*) *j* = 1,…*N*.

The crowded comparison is performed as described below [4, 46].

For any two query plans *qp*_1_ and *qp*_2_, *qp*_1_ is selected if (*qp*_1_≺_*n*_ *qp*_2_). *qp*_1_≺_*n*_ *qp*_2_ is true if either one of the following holds:

- **ρ**_rank⁡_(*qp*_1_)<**ρ**_rank⁡_(*qp*_2_)
- if *qp*_1_ and *qp*_2_ belong to the same front (i.e., if **ρ**_rank⁡_(*qp*_1_) = **ρ**_rank⁡_(*qp*_2_)), then *I*_*i*_(*d*_*qp*1_) > *I*_*i*_(*d*_*qp*2_).

**Step 6 (Crossover and Mutation)**

Crossover is performed on the selected query plans with a given crossover probability *P*_*c*_. It ensures proper exploration of the search space by combining the best features of the parent query plans (chromosomes). Mutation is performed on the given population with a given probability *P*_*m*_. It randomly changes the site (gene) in which the corresponding relation resides within a query plan (chromosome). The mutated gene always takes a random value from a set of valid sites for a particular relation. After going through the above steps, the first generation population is formed. NSGA-II follows a different method to produce subsequent generations in order to incorporate elitism as described next.

**Step 7 (Preserving Good Solutions (Elitism) [4])**

**Figure 2:**
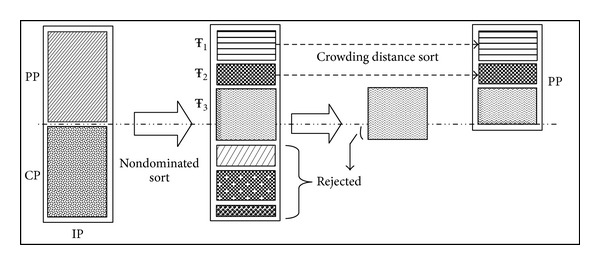
NSGA-II procedure preserving elitism [4].

In subsequent generations, the new population after each generation is combined with the parent population and a new intermediate population IP is created of size PP + CP, where PP is the parent population and CP is the child population as shown in Figure 2.

The non-dominated sort is applied to this intermediate population and fronts are formed as described in Step 3. Finally, the population for the next generation is formed by adding solutions from each front till the population size exceeds *P*. If the last front to be included was Ŧ_*c*_, which led to the population overflow, then query plans in Front Ŧ_*c*_ are selected based on their crowding distance measure (Step 4) in descending order until the population size exceeds *P*.

The above steps are repeated for “*G*” generations and the Top-*K* query plans are produced as output.

An example illustrating the use of the above NSGA-II based DQPG algorithm to generate query plans for a given distributed query is given next.

## 4. An Example

**Figure 3:**
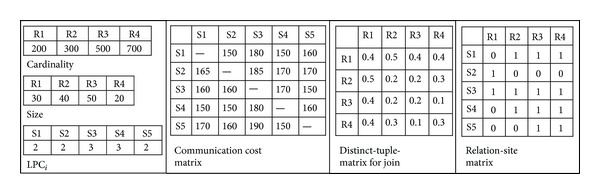
Matrices used for computing fitness [3].

Consider the site relation matrix, the communication-cost matrix, the local processing cost matrix, the distinct-tuple matrix, and the size matrix used to compute the fitness of query plans given in [3] and shown below in Figure 3. Suppose the initial parent population PP comprises of 10 query plans given in Table 1. Consider a query that accesses four relations (*R*1, *R*2, *R*3, and *R*4) which are distributed among five sites (*S*1, *S*2, *S*3, *S*4, and *S*5).

The computations of TPC and TCC for the query plan [2, 4, 1, 5] are given as follows.

(11) TPC4=(LPC2×a2)+(LPC4×a4) +(LPC5×a5)+(LPC1×a1),TCC4=(CC2,4×b2,4)+(CC4,5×b4,5)+(CC5,1×b5,1),

where

(12) LPC2×a2=0,CC2,4×b2,4=CC2,4×(Card(R1)×Size(R1))=170×(200×30)=1020000,LPC4×a4=LPC4×(Card(R1)×Card(R2)Dist1,2×Card(R1)×(Size(R1)+Size(R2)))=2×(200×3000.5×200×(70))=126000CC4,5×b4,5=CC4,5×(Card(R1)×Card(R2)Dist1,2×Card(R1)×(Size(R1)+Size(R2)))=170×(200×3000.5×200×(70))=6720000LPC5×a5 =LPC5  ×(Card(R1)×Card(R2)Dist1,2×Card(R1)×Card(R4)×(Dist2,4×Card(R1)×Card(R2)Dist1,2×Card(R1))−1×(Size(R1)+Size(R2)+Size(R4))) =2×(200×3000.5×200×7000.3×(200×300)/(0.5×200))  ×90=420000CC5,1×b5,1 =CC5,1  ×(Card(R1)×Card(R2)Dist1,2×Card(R1)×Card(R4)×(Dist2,4×Card(R1)×Card(R2)Dist1,2×Card(R1))−1×(Size(R1)+Size(R2)+Size(R4)))170×(200×3000.5×200×7000.3×(200×300)/(0.5×200))×90=35700000LPC1×a1 =LPC1×(Card(R1)  ×Card(R2)Dist1,2×Card(R1)×Card(R4)×(Dist2,4×Card(R1)×Card(R2)Dist1,2×Card(R1))−1×Card(R3)Dist4,3×Card(R3)×(∑i=14Size(Ri))) =2×(200×3000.5×200×7000.3×(200×300)/(0.5×200)×5000.1×500)×140=6533333.3.

So,

(13) $$\begin{array}{c} {\text{TPC}}_{4}=126000+420000+6533333.3=7079333.3\\ \text{TC}{\text{C}}_{4}=1020000+6720000+35700000=43440000. \end{array}$$

Similarly, TCC and TPC values of the other nine query plans are computed. Consider TCC and TPC of the 10 query plans are given in Table 2. The population is then sorted into different nondominated fronts as described in Step 3 of the proposed algorithm. For example, for query plan 1, that is, [2, 1, 5, 1], the set *S*_1_= {number of query plans that dominate query plan 1}. Since TCC [1] < TCC [4], TPC [1] < TPC [4], TCC [1] < TCC [10], and TPC [1] < TPC [10], the elements of *S*_1_ = {4,10}. Similarly the sets *S*_2_, …, *S*_10_ are computed and are given in Table 2. *n*_*i*_ stores the count of query plans that dominate *i*. So using the values in *S*_*i*_, *n*_1_ = 1, as only query plan 5 is dominating 1 and *n*_2_ = 1, as only query plan 3 is dominating 2. Similarly *n*_2_, …, *n*_10_ are computed and are given in Table 2. From Table 2, it can be noted that query plans 3 and 5 are not dominated as *n*_3_ = 0, *n*_5_ = 0. So, they are assigned to the first nondominated front Ŧ1. The elements in the next front are computed by reducing the count in *n*_*i*_ for each *i* ∈ *S*_3_ and *i* ∈ *S*_5_. So, *n*_*i*_ = {0, 0, − , 4, − , 0, 0, 1, 1, 7}. So the second front has query plans 1, 2, 6, and 7. This process continues till all the query plans in the population are assigned to their respective nondominated fronts. The fronts Ŧ_1_, Ŧ_2_, Ŧ_3_, and Ŧ_4_ are formed and are given in Table 2.

Finally the query plans are sorted separately on the values of TCC and TPC within each front as shown in Table 3.

After the population is sorted into different fronts, the crowding distance (*I*[*distance*]) computation is performed for each query plan using the formula given in Step  4 of the proposed algorithm. Query plans having the maximum and minimum values in each front are assigned *∞* distance values, that is, *I*[3], *I*[5], *I*[7], *I*[1], *I*[8], *I*[4], *I*[9], and *I*[10] are assigned *∞*. For the rest of the query plans, their crowding distance (CD) values are computed based on the sorted order of query plans in each front. Initially, they are assigned *I*[*distance*] = 0. For query plan 2 in front Ŧ_2_, the CD computation is performed as follows:

(14) Im1[2]=I[2]+TCC[6]−TCC[7]max⁡(TCC)−min⁡(TCC)=0+17020000−96000045090000−0=0.3562,I[2]=Im1[2]+TPC[7]−TPC[6]max⁡(TPC)−min⁡(TPC)=0.3562+6500000−384700010514000−2112000=0.6720.

CD of the query plans in the given population is given in Table 4.

Next, binary tournament selection is performed on the population on the basis of crowded comparison operator ≺_*n*_. This selection process is shown in Table 5.

The selected query plans undergo random single point crossover, with crossover probability *P*_*c*_ = 0.5. Mutation is performed on the selected population with mutation probability *P*_*m*_ = 0.02. The child population CP after crossover and mutation is shown in Table 6.

Now in accordance with NSGA-II algorithm, the populations from the second generation onward have to ensure elitism. For this purpose, the child population CP is combined with the parent population PP to generate intermediate population IP. This population is subjected to nondominated sort and fronts are formed as given in Table 7.

The population for the second generation is arrived at by selecting query plans based on front and within it based on crowding distance-wise, as described in Step 7, from the intermediate population IP till the actual population size 10 is exceeded. This selection is shown in Table 8.

The population PP for the second generation is given in Table 9.

The above process is repeated till a predefined number of generations *G* have elapsed. Thereafter, Top-*K* query plans are produced as output.

## 5. Experimental Results

The proposed NSGA-II based algorithm is implemented in MATLAB 7.7 in Windows 7 professional 64 bit OS, with Intel core i3 CPU at 2.13 GHz having 4 GB RAM. Experiments were carried out for a population of 100 query plans with each query plan involving 10 relations distributed over 50 sites. These were performed on four datasets, each comprising a different relation-site matrix. Graphs were plotted to observe change in average TC (ATC) with respect to generations and Top-*K* query plans for different pairs of crossover and mutation rates.

**Figure 4:**
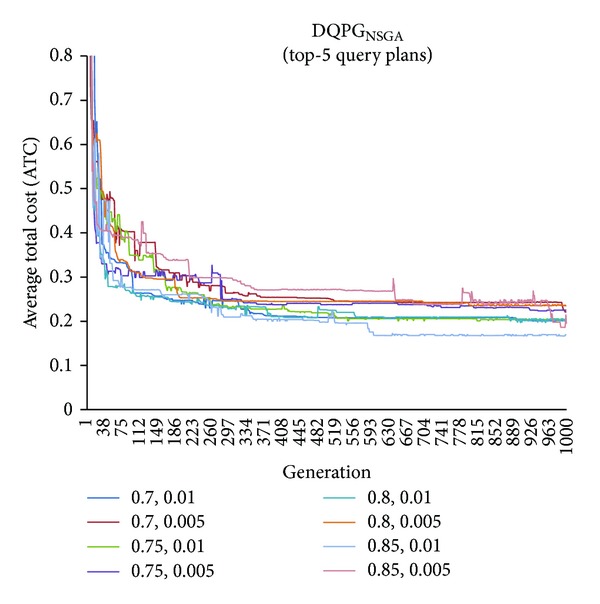


**Figure 5:**
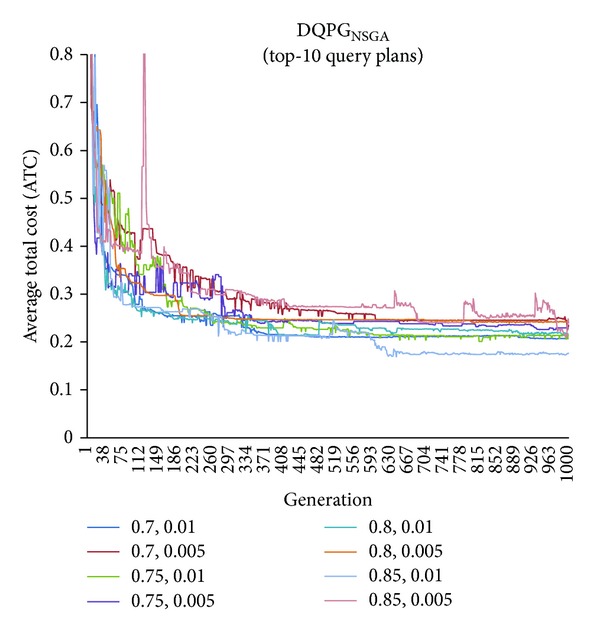


**Figure 6:**
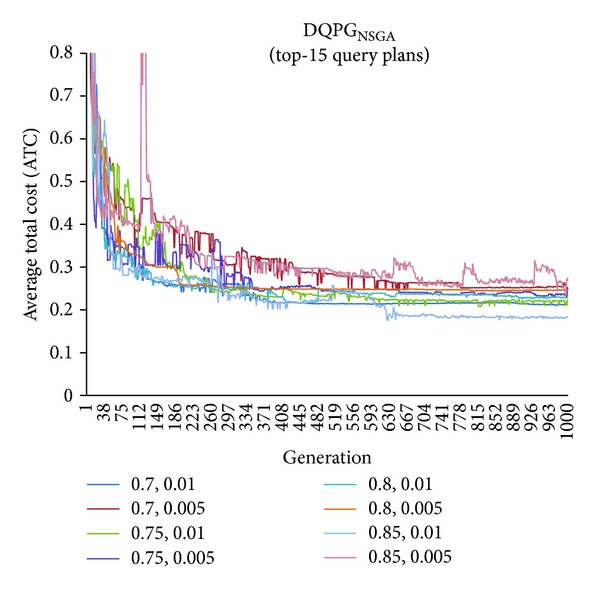


**Figure 7:**
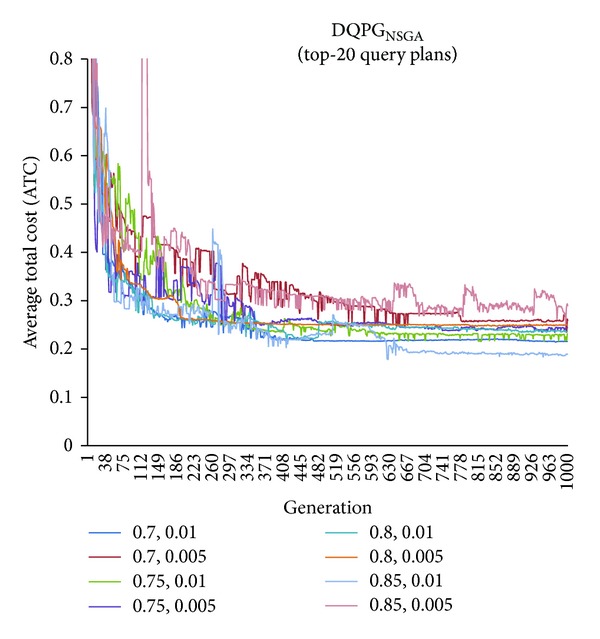


**Figure 8:**
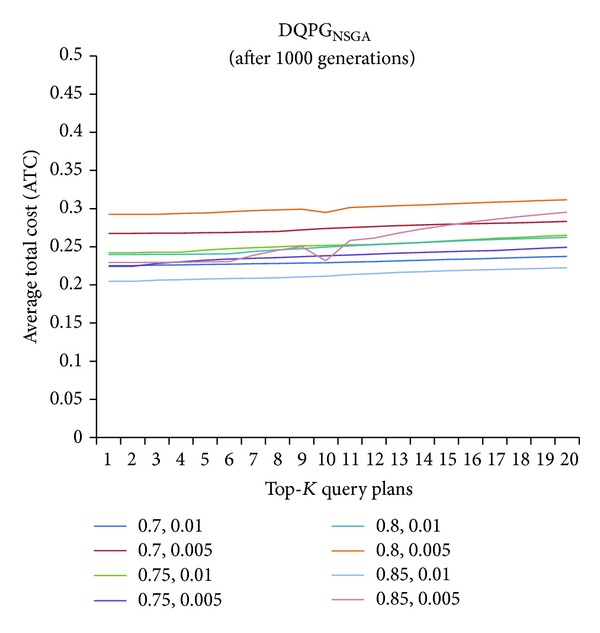


**Figure 9:**
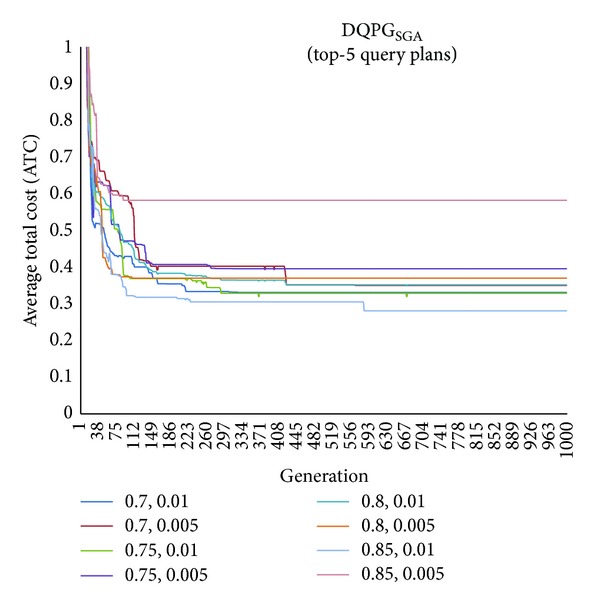


**Figure 10:**
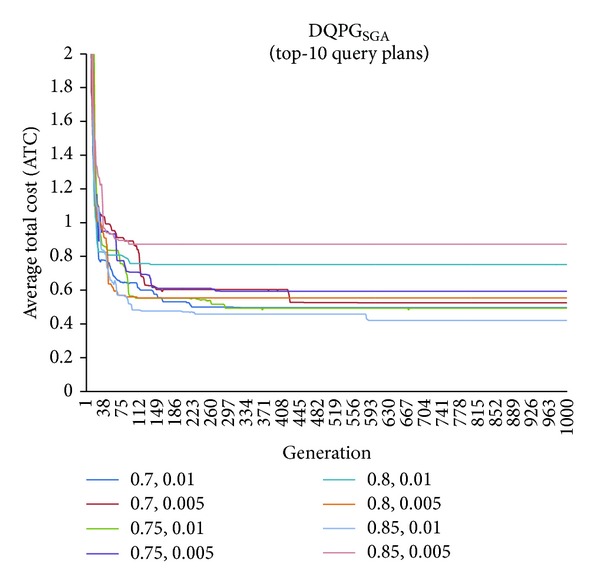


**Figure 11:**
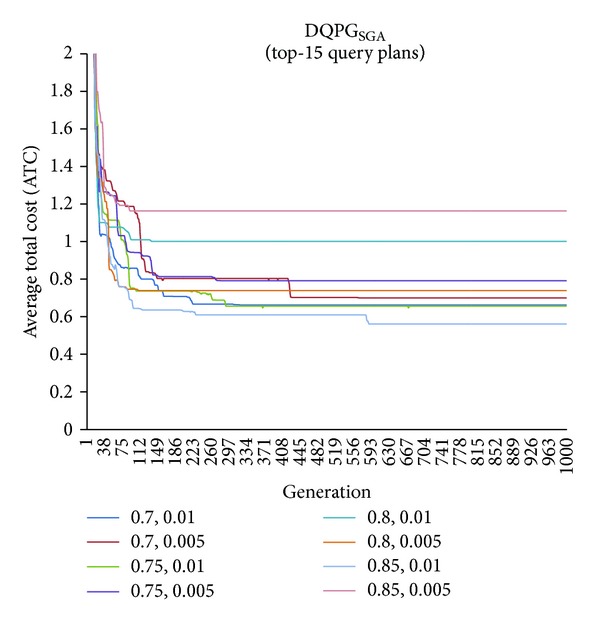


**Figure 12:**
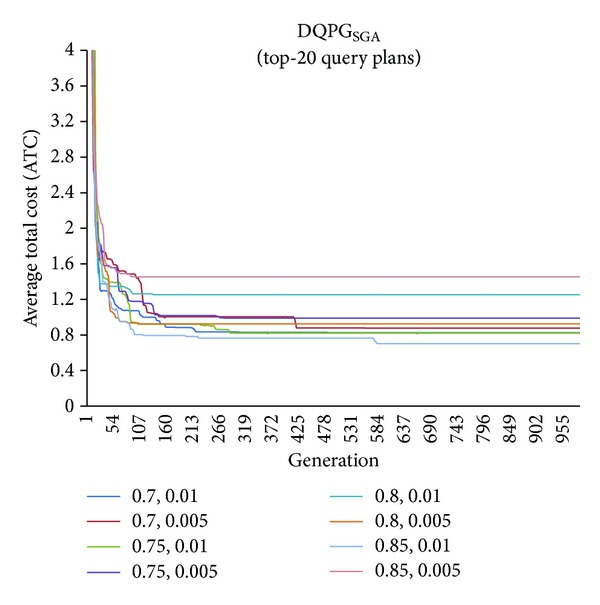


**Figure 13:**
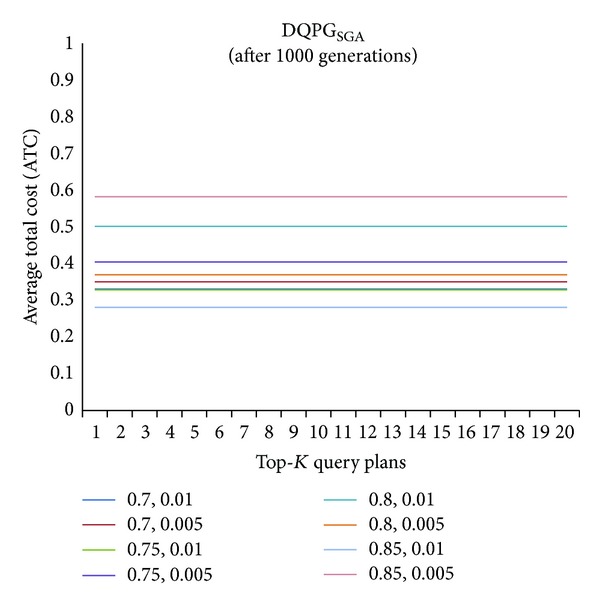


First, graphs showing the ATC values Top-5, Top-10, Top-15, and Top-20 query plans, averaged over four datasets, over 1000 generations for distinct pairs of crossover and mutation probability {*P*_*c*_, *P*_*m*_} = {0.7, 0.01}, {0.7, 0.005}, {0.75, 0.01}, {0.75, 0.005}, {0.8, 0.01}, {0.8, 0.005}, {0.85, 0.01}, {0.85, 0.005} using NSGA-II based DQPG algorithm (DQPG_NSGA_) were plotted. These are shown in Figures 4, 5, 6, and 7. It can be observed from these graphs that the convergence to ATC is lowest in the case of {*P*_*c*_, *P*_*m*_} = {0.85, 0.01}. Furthermore, the graph showing ATC values averaged over four datasets versus Top-*K* query plans after 1000 generations (Figure 8) also shows that the lowest ATC values achieved are for {*P*_*c*_, *P*_*m*_} = {0.85, 0.01}. Thus, it can be said that DQPG_NSGA_ performs reasonably well for {*P*_*c*_, *P*_*m*_} = {0.85, 0.01}. In order to compare DQPG_NSGA_ with SGA based DQPG algorithm (DQPG_NSGA_), similar graphs were plotted for DQPG_NSGA_. These are shown in Figures 9, 10, 11, 12, and 13. It is noted from these graphs that DQPG_NSGA_ also achieves convergence to the lowest ATC values for {*P*_*c*_, *P*_*m*_} = {0.85, 0.01}.

Since the two algorithms DQPG_NSGA_ and DQPG_NSGA_ converge to a lower ATC value for the same crossover and mutation probabilities, that is, {*P*_*c*_, *P*_*m*_} = {0.85, 0.01}, the comparisons of the two algorithms can be carried out for these observed probabilities.

**Figure 14:**
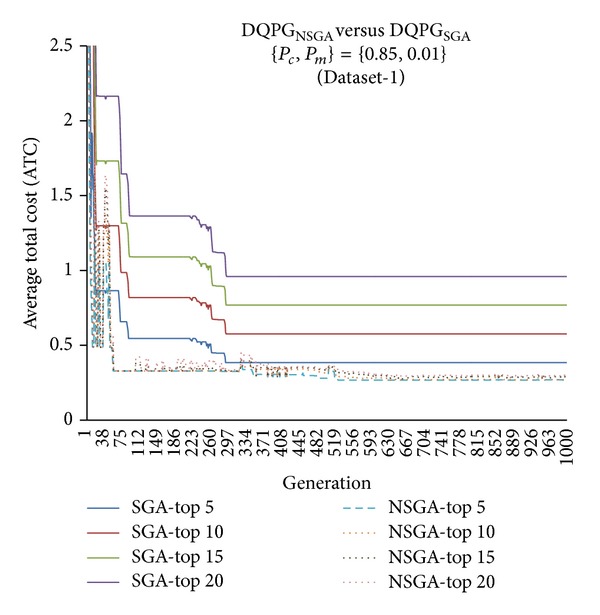


**Figure 15:**
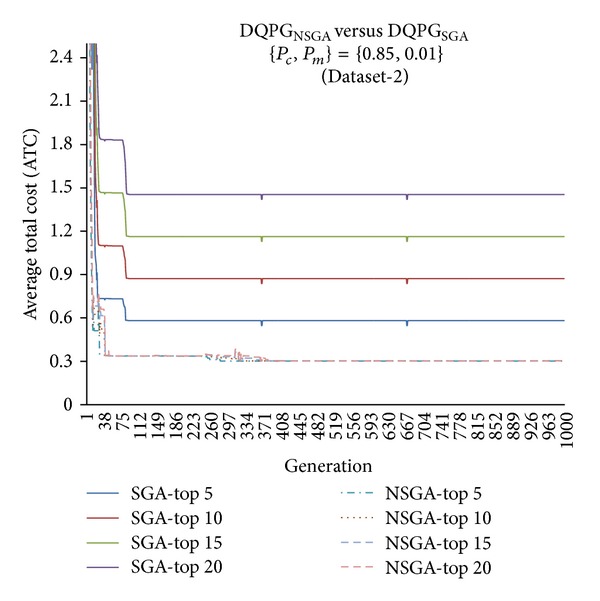


**Figure 16:**
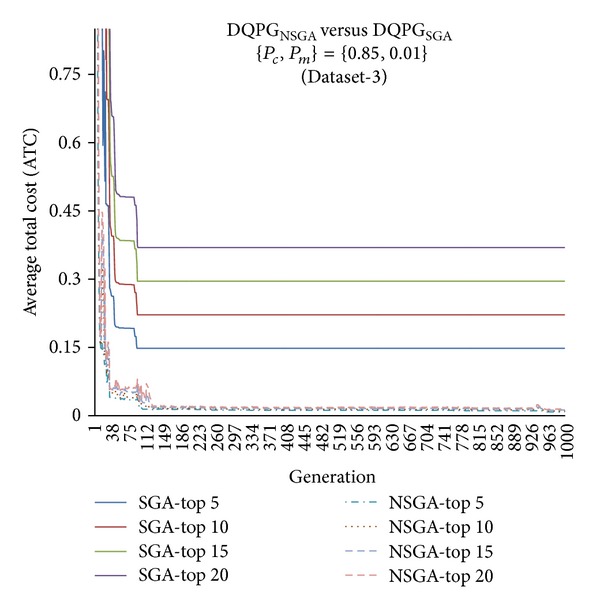


**Figure 17:**
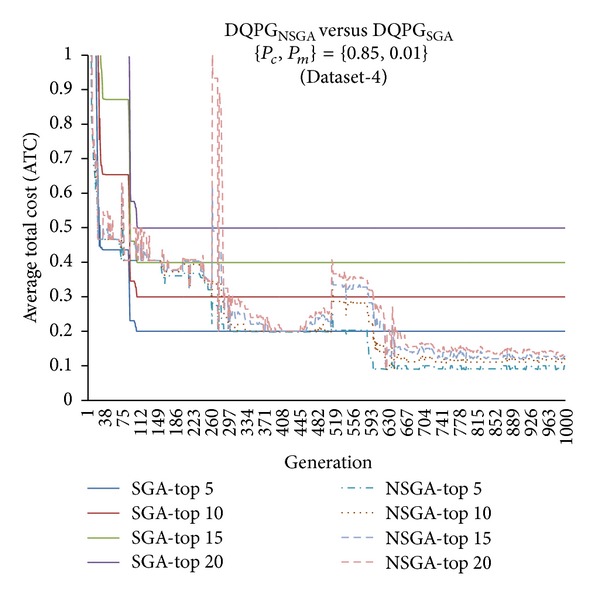


First, the two algorithms DQPG_NSGA_ and DQPG_NSGA_ were compared for each of the four datasets (Dataset-1, Dataset-2, Dataset-3, and Dataset-4) on the ATC values of Top-5, Top-10, Top-15, and Top-20 query plans generated over 1000 generations for {*P*_*c*_, *P*_*m*_} = {0.85, 0.01}. The corresponding graphs for each dataset are shown in Figures 14, 15, 16, and 17. It can be observed from the graphs that DQPG_NSGA_ converges to lower ATC values for Top-5, Top-10, Top-15, and Top-20 query plans generated for all datasets.

**Figure 18:**
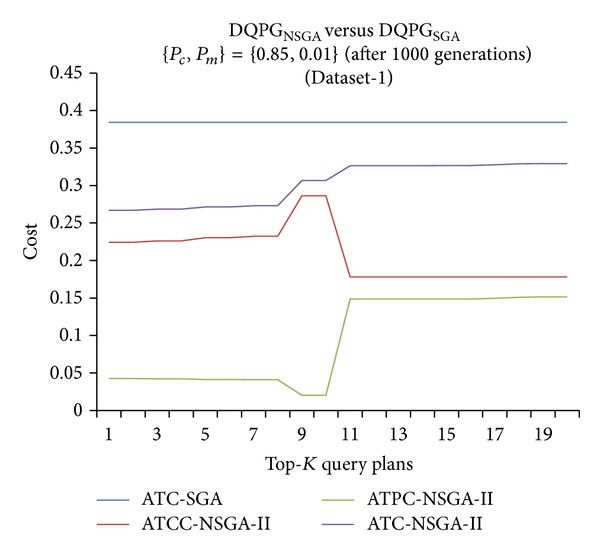


**Figure 19:**
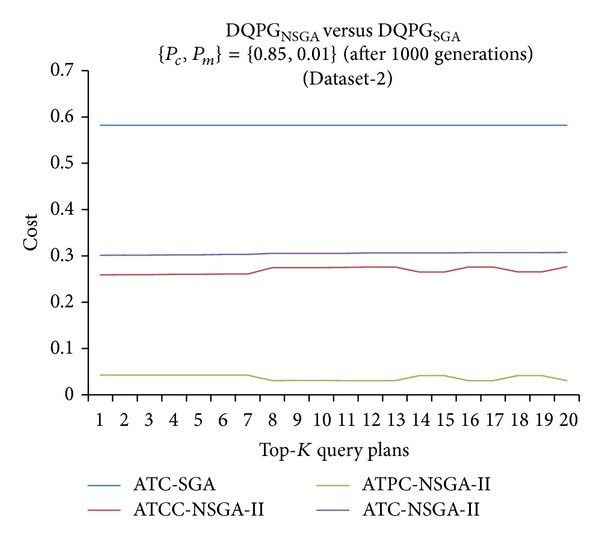


**Figure 20:**
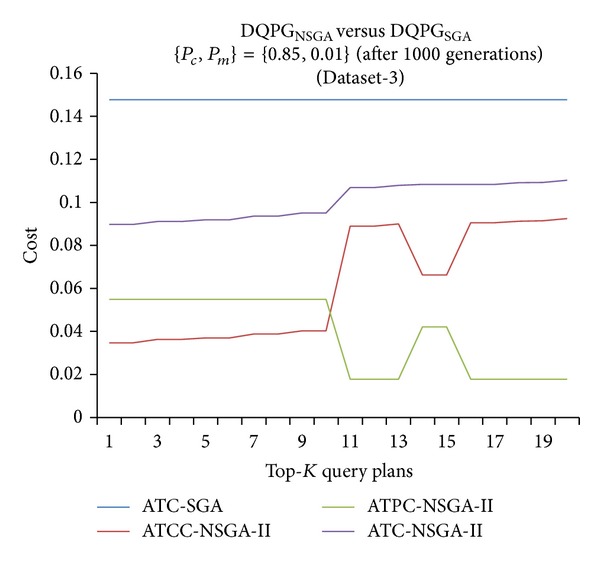


**Figure 21:**
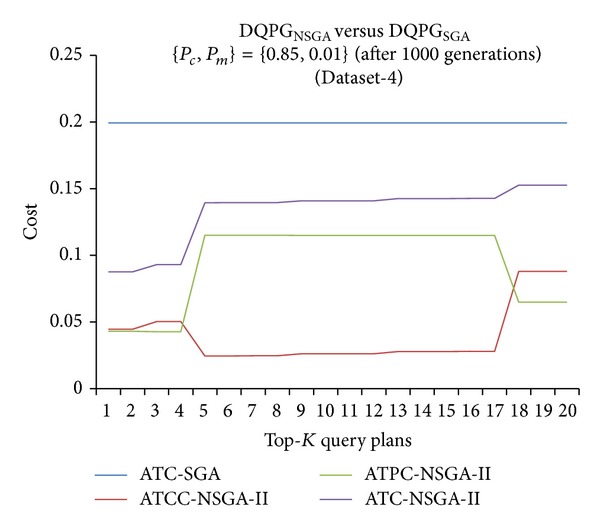


Furthermore, graphs comparing the ATC values of Top-*K* query plans produced by DQPG_NSGA_ and DQPG_NSGA_ after 1000 generations for the four datasets were plotted and are shown in Figures 18, 19, 20, and 21. These graphs also show average TCC (ATCC) and average TPC (ATPC) values of Top-*K* query plans generated by DQPG_NSGA_. It can be observed from these graphs that DQPG_NSGA_ is able to achieve an acceptable tradeoff between ATPC and ATCC, which in turn leads to a comparatively lower ATC for the Top-*K* query plans generated by it.

**Figure 22:**
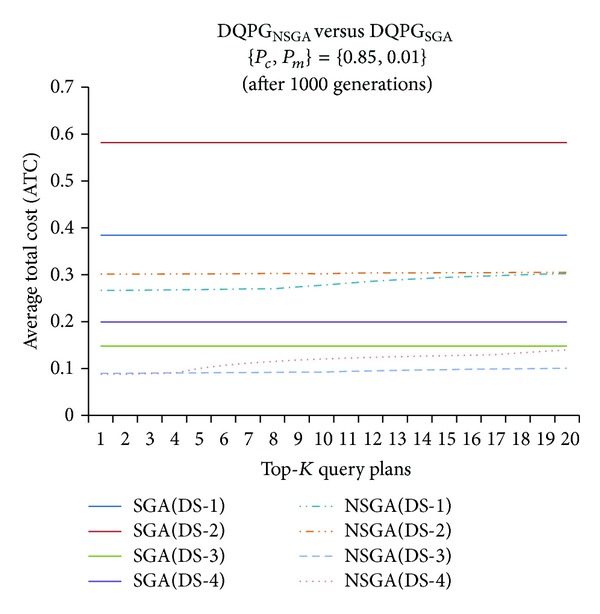


Next, a graph comparing the ATC values of Top-*K* query plans generated by DQPG_NSGA_ and DQPG_NSGA_ on all four datasets (DS-1, DS-2, DS-3, and DS-4) after 1000 generations for observed probabilities {*P*_*c*_, *P*_*m*_} = {0.85, 0.01} were plotted and is shown in Figure 22. It is noted from the graph that DQPG_NSGA_ performs better than DQPG_NSGA_ on the ATC values of Top-*K* query plans generated by the two algorithms for each of the four data sets.

It can be reasonably inferred from all the above graphs that DQPG_NSGA_ is able to generate Top-*K* query plans with lower ATC, when compared to those generated by DQPG_NSGA_. This may be attributed to acceptable tradeoffs achieved while simultaneously optimizing TPC and TCC, which results in lower TC in case of DQPG_NSGA_.

## 6. Conclusion

In this paper, DQPG problem given in [3] has been addressed, where query plans are generated for a distributed relational query that incurs minimum total query processing cost. Genetic algorithms have been used to generate these query plans. The total query processing cost TC in [3] can be viewed as comprising broadly of TPC and TCC, and therefore, minimizing TPC and TCC would result in minimizing TC. Thus, in this paper, the single-objective DQPG problem in [3] has been formulated and solved as a biobjective DQPG problem with the two objectives being minimizing TPC and minimizing TCC. These objectives are minimized simultaneously using the multiobjective genetic algorithm NSGA-II.

Experiments were performed and DQPG_NSGA_ is compared with DQPG_NSGA_ given in [3]. It was observed that both the algorithms individually gave good results for the crossover and mutation probabilities 0.85 and 0.01, respectively. The two algorithms were then compared on the ATC values of the Top-*K* query plans generated by them for the observed crossover and mutation probabilities. The results showed that DQPG_NSGA_ performed better than DQPG_NSGA_. Also the performance of the former was better when the two algorithms were compared on the ATC values of Top-*K* query plans. The better performance of DQPG_NSGA_ over DQPG_NSGA_ may be attributed to DQPG_NSGA_ achieving acceptable tradeoffs between TPC and TCC while minimizing TPC and TCC of Top-*K* query plans simultaneously.

## Figures and Tables

**Algorithm 1:**
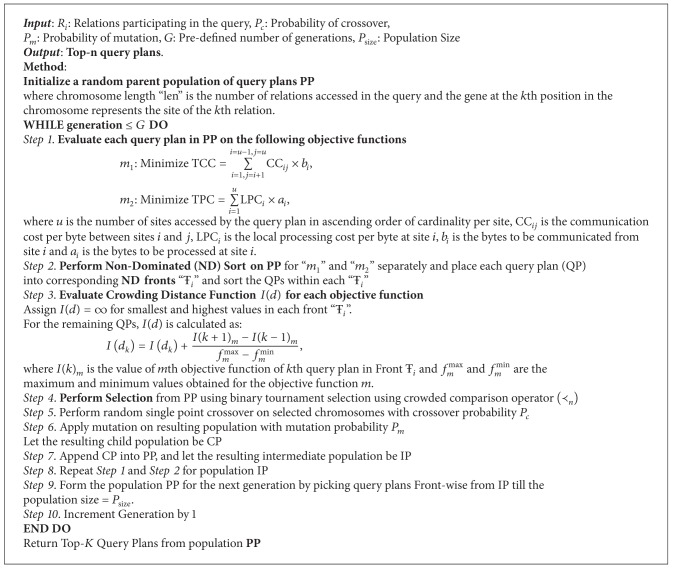
NSGA-II based DQPG algorithm.

**Table 1:** Initial parent population PP.

[2,1, 5,1]	[3,5, 4,5]
[3,3, 1,1]	[3,1, 1,1]
[3,3, 3,3]	[3,4, 4,4]
[2,4, 1,5]	[2,3, 3,3]
[3,1, 1,3]	[2,1, 3,4]

**Table 2:** Nondominated sort on PP.

Index [*i*]	Population PP	*m*_1_ = TCC	*m*_2_ = TPC	*S*_*i*_	*n*_*i*_	Front
[1]	[2,1, 5,1]	18020000	3746700	[4,10]	1	Ŧ_2_
[2]	[3,3, 1,1]	6720000	6006000	[4,10]	1	Ŧ_2_
[3]	[3,3, 3,3]	0	4200000	[2,4, 7,8, 9,10]	—	Ŧ_1_
[4]	[2,4, 1,5]	43440000	7079333.3	[10]	6	Ŧ_3_
[5]	[3,1, 1,3]	14000000	2112000	[1,4, 6,10]	—	Ŧ_1_
[6]	[3,5, 4,5]	17020000	3847000	[4,10]	1	Ŧ_2_
[7]	[3,1, 1,1]	960000	6500000	[4,8, 9,10]	1	Ŧ_2_
[8]	[3,4, 4,4]	1020000	9750000	[10]	2	Ŧ_3_
[9]	[2,3, 3,3]	1110000	9750000	[10]	2	Ŧ_3_
[10]	[2,1, 3,4]	45090000	10514000	—	9	Ŧ_4_

**Table 3:** Sorted fronts based on TCC and TPC.

Query plans sorted on *m*_1_ = TCC					Query plans sorted on *m*_2_ = TPC				
Ŧ_1_	3	5			Ŧ_1_	5	3		
Ŧ_2_	7	2	6	1	Ŧ_2_	1	6	2	7
Ŧ_3_	8	9	4		Ŧ_3_	4	8	9	
Ŧ_4_	10				Ŧ_4_	10			

**Table 4:** Results for the objective functions on PP.

Index [*i*]	Population PP	*m*_1_ = TCC	*m*_2_ = TPC	Front	Crowding distance (CD) = *I*[*i*]
[1]	[2,1, 5,1]	18020000	3746700	Ŧ_2_	∞
[2]	[3,3, 1,1]	6720000	6006000	Ŧ_2_	0.672
[3]	[3,3, 3,3]	0	4200000	Ŧ_1_	∞
[4]	[2,4, 1,5]	43440000	7079333.3	Ŧ_3_	∞
[5]	[3,1, 1,3]	14000000	2112000	Ŧ_1_	∞
[6]	[3,5, 4,5]	17020000	3847000	Ŧ_2_	0.5195
[7]	[3,1, 1,1]	960000	6500000	Ŧ_2_	∞
[8]	[3,4, 4,4]	1020000	9750000	Ŧ_3_	∞
[9]	[2,3, 3,3]	1110000	9750000	Ŧ_3_	∞
[10]	[2,1, 3,4]	45090000	10514000	Ŧ_4_	∞

**Table 5:** Selection of query plans using binary tournament selection technique.

Index	Randomly generated indexes [*i*] and [*j*]	Tournament between query plans [*P*(*i*)] and [*P*(*j*)]	Front comparison	Query plan selected (lower front or higher CD)
[1]	[1] and [4]	[2,1, 5,1] and [2,4, 1,5]	[Ŧ_2_] and [Ŧ_3_]	[2,1, 5,1]
[2]	[2] and [3]	[3,3, 1,1] and [3,3, 3,3]	[Ŧ_2_] and [Ŧ_1_]	[3,3, 3,3]
[3]	[3] and [4]	[3,3, 3,3] and [2,4, 1,5]	[Ŧ_1_] and [Ŧ_3_]	[3,3, 3,3]
[4]	[2] and [6]	[3,3, 1,1] and [3,5, 4,5]	[Ŧ_2_] and [Ŧ_2_]	[3,3, 1,1]
[5]	[2] and [4]	[3,3, 1,1] and [2,4, 1,5]	[Ŧ_2_] and [Ŧ_3_]	[3,3, 1,1]
[6]	[1] and [3]	[2,1, 5,1] and [3,3, 3,3]	[Ŧ_2_] and [Ŧ_1_]	[3,3, 3,3]
[7]	[2] and [5]	[3,3, 1,1] and [3,1, 1,3]	[Ŧ_2_] and [Ŧ_1_]	[3,1, 1,3]
[8]	[2] and [8]	[3,3, 1,1] and [3,1, 1,3]	[Ŧ_2_] and [Ŧ_3_]	[3,3, 1,1]
[9]	[7] and [8]	[3,1, 1,1] and [3,1, 1,3]	[Ŧ_2_] and [Ŧ_3_]	[3,1, 1,1]
[10]	[2] and [9]	[3,3, 1,1] and [2,4, 1,5]	[Ŧ_2_] and [Ŧ_3_]	[3,3, 1,1]

**Table 6:** Population CP after crossover and mutation.

Index	Population after crossover	Population after mutation (CP)
[1]	[2,1, 1,1]	[2,1, 1,1]
[2]	[3,3, 1,1]	[3,3, 1,1]
[3]	[3,3, 5,1]	[**3, 3, *4*, 1**]
[4]	[3,3, 1,1]	[3,3, 1,1]
[5]	[3,3, 3,1]	[3,3, 3,1]
[6]	[3,3, 3,3]	[3,3, 3,3]
[7]	[3,1, 1,3]	[3,1, 1,3]
[8]	[3,3, 1,3]	[3,3, 1,3]
[9]	[3,1, 3,3]	[3,3, 3,3]
[10]	[3,3, 1,1]	[3,3, 1,1]

**Table 7:** Front assignment for query plans in IP.

Index	Intermediate population IP	TCC	TPC	Front
[1]	[2,1, 5,1]	18020000	3747000	Ŧ_2_
[2]	[3,3, 1,1]	6720000	6006000	Ŧ_3_
[3]	[3,3, 3,3]	0	4200000	Ŧ_1_
[4]	[2,4, 1,5]	43440000	7079000	Ŧ_4_
[5]	[3,1, 1,3]	14000000	2112000	Ŧ_1_
[6]	[3,5, 4,5]	17020000	3847000	Ŧ_2_
[7]	[3,1, 1,1]	960000	6500000	Ŧ_2_
[8]	[3,1, 1,3]	1020000	9750000	Ŧ_3_
[9]	[2,3, 3,3]	1110000	9750000	Ŧ_3_
[10]	[2,1, 3,4]	45090000	10514000	Ŧ_5_
[11]	[2,1, 1,1]	990000	6500000	Ŧ_2_
[12]	[3,3, 1,1]	6720000	6006000	Ŧ_3_
[13]	[3,3, 4,1]	90300000	8946000	Ŧ_5_
[14]	[3,3, 1,1]	6720000	6006000	Ŧ_3_
[15]	[3,3, 3,1]	2520000	4230000	Ŧ_2_
[16]	[3,3, 3,3]	0	4200000	Ŧ_1_
[17]	[3,1, 1,3]	14000000	2112000	Ŧ_1_
[18]	[3,3, 1,3]	4500000	2640000	Ŧ_1_
[19]	[3,3, 3,3]	0	4200000	Ŧ_1_
[20]	[3,3, 1,1]	6720000	6006000	Ŧ_3_

**Table 8:** Nondominated sorting for query plans in IP.

Index	Intermediate population IP	Front	Crowding distance
[3]	[3,3, 3,3]	Ŧ_1_	
[5]	[3,1, 1,3]	Ŧ_1_	
[16]	[3,3, 3,3]	Ŧ_1_	
[17]	[3,1, 1,3]	Ŧ_1_	
[18]	[3,3, 1,3]	Ŧ_1_	
[19]	[3,3, 3,3]	Ŧ_1_	
[1]	[2,1, 5,1]	Ŧ_2_	∞
[7]	[3,1, 1,1]	Ŧ_2_	∞
[11]	[2,1, 1,1]	Ŧ_2_	∞
[15]	[3,3, 3,1]	Ŧ_2_	0.4933

[6]	[3,5, 4,5]	Ŧ_2_	0.2292
[2]	[3,3, 1,1]	Ŧ_3_	
[9]	[2,3, 3,3]	Ŧ_3_	
[8]	[3,1, 1,3]	Ŧ_3_	
[12]	[3,3, 1,1]	Ŧ_3_	
[14]	[3,3, 1,1]	Ŧ_3_	
[20]	[3,3, 1,1]	Ŧ_3_	
[4]	[2,4, 1,5]	Ŧ_4_	
[10]	[2,1, 3,4]	Ŧ_5_	
[13]	[3,3, 4,1]	Ŧ_4_	

**Table 9:** Population PP in the 2nd generation.

Index	Population PP
[1]	[3,3, 3,3]
[2]	[3,1, 1,3]
[3]	[3,3, 3,3]
[4]	[3,1, 1,3]
[5]	[3,3, 1,3]
[6]	[3,3, 3,3]
[7]	[2,1, 5,1]
[8]	[3,1, 1,1]
[9]	[2,1, 1,1]
[10]	[3,3, 3,1]
